# Construction of a high-density genetic map and dissection of genetic architecture of six agronomic traits in tobacco (*Nicotiana tabacum* L.)

**DOI:** 10.3389/fpls.2023.1126529

**Published:** 2023-02-15

**Authors:** Zhijun Tong, Manling Xu, Qixin Zhang, Feng Lin, Dunhuang Fang, Xuejun Chen, Tianneng Zhu, Yingchao Liu, Haiming Xu, Bingguang Xiao

**Affiliations:** ^1^ Key Laboratory of Tobacco Biotechnological Breeding, National Tobacco Genetic Engineering Research Center, Yunnan Academy of Tobacco Agricultural Sciences, Kunming, Yunnan, China; ^2^ Institute of Bioinformatics and Institute of Crop Science, College of Agriculture and Biotechnology, Zhejiang University, Hangzhou, Zhejiang, China

**Keywords:** tobacco, linkage map, mixed linear model, epitasis, genotype-by-environment interactions

## Abstract

Tobacco (*Nicotiana tabacum* L.) is an economic crop and a model organism for studies on plant biology and genetics. A population of 271 recombinant inbred lines (RIL) derived from K326 and Y3, two elite flue-cured tobacco parents, has been constructed to investigate the genetic basis of agronomic traits in tobacco. Six agronomic traits including natural plant height (nPH), natural leaf number (nLN), stem girth (SG), inter-node length (IL), length of the largest leaf (LL) and width of the largest leaf (LW) were measured in seven environments, spanning the period between 2018 and 2021. We firstly developed an integrated SNP-indel-SSR linkage map with 43,301 SNPs, 2,086 indels and 937 SSRs, which contained 7,107 bin markers mapped on 24 LGs and covered 3334.88 cM with an average genetic distance of 0.469cM. Based on this high-density genetic map, a total of 70 novel QTLs were detected for six agronomic traits by a full QTL model using the software *QTLNetwork*, of which 32 QTLs showed significant additive effects, 18 QTLs showed significant additive-by-environment interaction effects, 17 pairs showed significant additive-by-additive epistatic effects and 13 pairs showed significant epistasis-by-environment interaction effects. In addition to additive effect as a major contributor to genetic variation, both epistasis effects and genotype-by-environment interaction effects played an important role in explaining phenotypic variation for each trait. In particular, *qnLN6-1* was detected with considerably large main effect and high heritability ( $h_{a}^{2}$ =34.80%). Finally, four genes including *Nt16g00284.1*, *Nt16g00767.1*, *Nt16g00853.1*, *Nt16g00877.1* were predicted as pleiotropic candidate genes for five traits.

## 1 Introduction

Tobacco (*Nicotiana tabacum* L.) is an important economic crop widely grown throughout the temperate agricultural zones in the world. According to FAO-statistics, approximately 5.9 million tons of tobacco were produced worldwide in 2020, and China accounted for 36.3% of the total yields (Food and Agriculture Organization of the United Nations, 2020). Tobacco products are mainly derived from leaves, which are post-processed and consumed as cigarettes, cigars, pipes, and chewing tobacco, and cultivating high-yield and high-quality tobacco leaves will increase the economic value of the tobacco industry (Wu et al., 2020). Therefore, genetic improvements of the agronomic traits related with leaves are of great importance for tobacco industry. In addition to being an essential crop in agriculture, tobacco is also a typical model plant for genetic study, pathology investigation and biotechnology (Liu et al., 2013; Sun et al., 2015; Wang and Bennetzen, 2015; Wang and Balint-Kurti, 2016; Xiu et al., 2016; Bao et al., 2017; Pagliano et al., 2017; Lee and Kim, 2018). However, the genetic mechanisms underlying important agronomic traits in tobacco are not well-characterized owing to the complexity of genome and limitation of molecular genetic resources (Julio et al., 2006; Lewis et al., 2007).

As a member of the family Solanaceae, tobacco is an allotetraploid originated from two ancestral parents *Nicotiana sylvestris* (2n = 24) and *Nicotiana tomentosiformis* (2n = 24) about 200,000 years ago (Leitch et al., 2008; Sierro et al., 2013; Sierro et al., 2014). The genome of tobacco is massive (~4.5 Gb) and complex, with numerous repetitive sequences (Zimmerman and Goldberg, 1977). Advances in sequencing technologies and genome assembly methods have allowed for the generation of a nearly complete reference genome for allotetraploid tobacco (Sierro et al., 2013; Sierro et al., 2014; Edwards et al., 2017; Thimmegowda et al., 2018). In addition, efforts to develop more molecular genetic markers, from microsatellites or simple sequence repeats (SSRs) to single nucleotide polymorphisms (SNPs), have made major achievements, which enabled the construction of high-density linkage maps (Bindler et al., 2011; Tong et al., 2012; Xiao et al., 2015; Gong et al., 2016; Tong et al., 2016; Thimmegowda et al., 2018; Tong et al., 2020). For example, Xiao et al. (2015) developed 4138 SNP markers using next-generation RAD sequencing and constructed a genetic map covering a total length of 1944.74 cM with a population of 193 backcross individuals. Recently, a genetic map with 45,081 SNPs has been constructed by whole genome sequencing using a tobacco population including 274 accessions (Tong et al., 2020). However, construction of a higher density genetic map is still necessary and valuable for more in-depth genetic dissection of complex traits. Therefore, based on the same population, we discovered more polymorphic markers, including indels (insertions or deletions) and SSRs, to the published SNPs (Tong et al., 2020), and constructed an integrated linkage map. This higher-density linkage map will provide a more solid foundation for fine mapping of quantitative trait locus (QTL), further functional analysis by bioinformatics tools and dissection of complicate genetic architecture in tobacco.

Because of high-density and high-quality genetic maps unavailable, QTL mapping studies in tobacco have lagged behind many other crops, including maize (Liu et al., 2014; Zhang et al., 2017), rice (Dixit et al., 2017; Hu et al., 2018), and wheat (Prat et al., 2017; Tura et al., 2020). Up to now, the conducted QTL mapping studies in tobacco were mainly related to the traits diseases resistance (e.g. black shank, brown spot and soilborne disease) (Tong et al., 2012; Drake-Stowe et al., 2017; Zhang et al., 2018; Cheng et al., 2019; Ma et al., 2019), agronomic performance (e.g. various morphological traits) (Lewis et al., 2007; Wu et al., 2014; Cheng et al., 2015; Tong et al., 2021; Liu et al., 2022) and leaf quality (e.g. chemical components and smoke properties) (Julio et al., 2006; Vijay et al., 2010). Particularly, for important agronomic traits associated with yield, few QTLs of tobacco have been reported so far (Lewis et al., 2007; Cheng et al., 2015; Tong et al., 2021; Liu et al., 2022). Moreover, those studies of QTL mapping mainly focused on the QTL effects, epistatic effects were barely investigated as well as their interactions with environments. However, epistasis has long been recognized to be fundamentally important for revealing genetic architecture of quantitative traits, identification and estimation of interaction effects of epistatic loci will benefit to improve predictions of heterosis in crop species (Mackay, 2014). In further, it has been known that the magnitude of gene effects is subjected to the difference of environments, that is depicted as interaction of gene by environment, which plays an important role in determining the environmental adaptability and genetic stability of varieties (Wang et al., 2014); Therefore, exploring the environment-specific effects of QTL is indispensable in QTL study on complex traits.

In this study, we developed a new integrated SNP-indel-SSR linkage map for a population of tobacco RILs derived from K326 and Y3 with 274 individuals. Based on the high-density linkage map and multi-environment phenotypic data of the RIL population, QTL mapping was conducted for six agronomic traits; the detected main-effect QTLs, epistasis QTLs and their interactions with environments provided more insights into the genetic architecture of the traits; these results will greatly facilitate the molecular improvements of breeding target traits in tobacco.

## 2 Materials and methods

### 2.1 Plant materials and field trial

The recombinant inbred lines (RILs) were generated from two elite flue-cured tobacco parents Y3 and K326. Y3 is a backbone cultivated variety that originated from Zimbabwe with elite agronomic traits and complicated parental sources. K326, whose genome has been assembled (Edwards et al., 2017), was introduced from America with high commercial quality and disease resistance but moderate agronomic performance. A total of 274 genotypes were employed in this study, consisting of two parents, one F_1_ generation individual (YKF_1_; Y3 × K3K6) and 271 F_7_ generation individuals. The materials were planted at Yanhe (N: 24.35; E: 102.54) and Shilin (N: 23.46; E: 103.17) field experiment stations using complete random design with 5 replications, and were cultivated according to local technical measures for quality tobacco production. Six agronomic traits, including natural plant height (nPH), natural leaf number (nLN), stem girth (SG), inter-node length (IL), length of the largest leaf (LL) and width of the largest leaf (LW), were measured in 2018, 2019, 2020 and 2021 years in Yanhe, and in 2018, 2019 and 2020 in Shilin. Seven different combinations of location and year were treated as environments denoted as E1 (2018 Shilin), E2 (2018 Yanhe), E3 (2019 Shilin), E4 (2019 Yanhe), E5 (2020 Shilin), E6 (2020 Yanhe) and E7 (2021 Shilin). Six agronomic traits were investigated on the day 65 after planting in the field (first green fruit stage) according to the tobacco industry standard YC/T 369-2-10.

### 2.2 Statistical analysis of phenotypes

Variance components analysis and heritability estimation were performed based on the following mixed linear model,


$$y_{khi}=\mu +g_{k}+e_{h}+ge_{kh}+\epsilon_{khi}$$

where *y_khi_* is the phenotypic value of the *i*-th replication of the *k*-th individual in the *h*-th environment; *μ* is the population mean; *g_k_* is the genotypic value of the *k*-th genotype, random effect, subjected to a normal distribution $g_{k}\sim N\left(0,\sigma_{g}^{2}\right)$ ; *ge_kh_* the effect of the *h*-th environment, random, subjected to the normal distribution $e_{h}\sim N\left(0,\sigma_{e}^{2}\right)$ ; *ge_kh_* the interaction effect between the *k*-th genotype and the *h*-th environment, subjected to the normal distribution $ge_{kh}\sim N\left(0,\sigma_{ge}^{2}\right)$ , random effect; *ε_khi_* is the residual effect of the individual, random, $\epsilon_{khi}\sim N\left(0,\sigma_{\epsilon }^{2}\right)$ . The *mmer* module of *sommer* R package (Covarrubias-Pazaran, 2016) was applied to estimate the variances of random effects ( ${\hat{\sigma }}_{g}^{2},{\hat{\sigma }}_{e}^{2},\;{\hat{\sigma }}_{ge}^{2},{\hat{\sigma }}_{\epsilon }^{2}$ ) and to predict the random effects by BLUPs (best linear unbiased predictions, ${\hat{g}}_{k},{\hat{e}}_{h},{\hat{ge}}_{kh}$ ) by solving the mixed model equation (MME). Broad sense heritability was estimated with the formula ${\hat{H}}^{2}={\hat{\sigma }}_{g}^{2}/\left({\hat{\sigma }}_{g}^{2}+{\hat{\sigma }}_{ge}^{2}+{\hat{\sigma }}_{\epsilon }^{2}\right)$ , there ${\hat{\sigma }}_{g}^{2}$ was the estimated genotypic variance, $\;{\hat{\sigma }}_{e}^{2}$ was the estimated environmental variance, ${\hat{\sigma }}_{ge}^{2}$ was the estimated variance due to genotype-by-environment interaction, and $\;{\hat{\sigma }}_{\epsilon }^{2}$ was the estimated residual variance. The *rcorr* module of *Hmisc* R package (https://cran.r-project.org/web/packages/Hmisc/index.html) was employed to calculate Pearson correlation coefficients between six studied traits: (1) phenotypic correlation coefficients with *y_khi_* for each environment, respectively; (2) genetic correlation coefficients with ${\hat{y}}_{k}$ . ( ${\hat{y}}_{k}=\hat{\mu }+{\hat{g}}_{k}$ ), where ${\hat{y}}_{k}$ is the corrected genotypic value of the *k*-th individual by population mean, $\hat{\mu }$ is the estimated population mean, and ${\hat{g}}_{k}$ is the genotypic value of the *k*-th individual predicted by BLUP.

### 2.3 DNA extraction and sequencing

The genomic DNA of 274 samples (271 RIL F_7_ individuals, the parents, and one F_1_ generation) was extracted from the leaves of 7-week-old tobacco seedlings by Qiagen DNeasy Plant Mini Kits (Qiagen, Hilden, Germany) and sonicated into ~350 bp fragments *via* a Covaris M220 system (Covaris, Woburn, MA. USA). The paired end DNA fragments were used to generate DNA Nano Balls *via* LM-PCR and rolling circle amplification. The sequencing library was constructed according to the requirements of the BIGSEQ-500 platform and sequenced with PE100 (Li et al., 2018).

### 2.4 Reads filtering and mapping

The raw data were filtered by SOAPnuke (v1.5.6) with the default parameters except “-l 40 -q 0.2”. The genome of Nicotiana tabacum (K326 cultivar) was used as a reference genome (Edwards et al., 2017). After eliminating the scaffolds that were shorter than 500 bp, there were 942,190 scaffolds (~ 4.6Gb) retained for analysis. The clean reads were mapped to the reference using the “mem” method in BWA software (−t 8 -k 19 -M –R) (Li, 2013). The sam files were transferred to sortbam *via* SortSam.jar in pi- card software (“Picard Toolkit.” 2019. Broad Institute, GitHub Repository. http://broadinstitute.github.io/picard/).

### 2.5 SNP/Indel calling and filtering, SSR marker resources

SNPs and indels were called using GATK (GATK3.3.0) and filtered according to the following criteria (McKenna et al., 2010): For SNPs, (1) SNPs of the parents and F_1_ generation were filtered based on DP ≥10 and GQ ≥25. (2) SNPs of the 271 RIL F_7_ individuals were filtered based on DP ≥3. (3) SNPs that were absent in the parents and F_1_ generation were removed. (4) SNPs that exhibited a missing rate of over 20% in the 271 RIL F_7_ individuals were eliminated. (5) Triallelic or tetraallelic sites were deleted. For indels, (1) Indels of the parents and F_1_ generation were filtered based on DP ≥10 and GQ ≥40. (2) Indels of the 271 RIL F_7_ individuals were filtered based on DP ≥3 and GQ ≥10. (3) Indels that were absent in the parents and F_1_ generation, were removed. (4) Indels that exhibited a missing rate of over 20% in the 271 RIL F_7_ individuals were eliminated. (5) Triallelic or tetraallelic sites were deleted. (6) Indels over 5bp were removed. After quality controls, there were 10,057,282 high-quality SNPs and 569,946 indels remained for subsequent analysis. Then, we screened out 1,626,811 SNPs and 71,130 indels which exhibited two different homozygous genotypes in two parents (aa and bb) and heterozygous (ab) in F_1_ (hereinafter referred to as aa × bb & F_1_-h types); consequently, high quality linkage map will be guaranteed after eliminating the influence of uncertainty in genotype because of the high heterozygosity of Y3.

A total of 937 SSRs were provided by Tong et al. (2016) and the primer pairs of SSRs were presented in the **Supplementary Table 1**.

### 2.6 Genetic linkage map

The number of the markers (1,626,811 SNPs and 71,130 indels) that were aa × bb & F_1_-h types was over whelming for most software used to construct genetic linkage maps. Therefore, we clustered the markers that mapped on the same scaffold, kept one marker per 100 Kb as the low recombination rate within a short physical distance, and selected out 44,804 SNPs and 2,163 indels. Together with 937 published SSRs, a total of 47,904 markers were selected uniformly for genetic linkage map construction in software LepMap3 (Rastas, 2017). In detail, 47,814 markers were identified as informative *via* the ParentCall2 module, and anchored to 24 LGs using the SeparateChromsomes2 module (LOD = 83). The genetic distance of each LG was calculated by the OrderMarkers2 module. The genetic linkage density map was visualized using R package in LinkageMapView (Ouellette et al., 2018).

### 2.7 Genetic model and statistical methods for QTL mapping

A saturated genetic model was adopted for modeling the genetic architecture of complex traits from multi-environment trials, which includes additive effect (*a*) of each QTL, additive-by-additive epistatic effect (*aa*) of each paired QTLs, treated as fixed effects, and their corresponding environment interaction effects (*ae* and *aae*) as random effects. Suppose a trait is controlled by *s* segregating QTL in which *t* pair of QTL involved interaction. Then, the phenotypic value of the *m*-th replication of the *k*-th genotypes in the *h*-th environment (*y_hkm_*) can be expressed by the following mixed linear model,


$$\begin{array}{c} y_{hkm}=\mu +\sum_{i=1}^{s}a_{i}x_{ik}+\sum_{i,j\in \left\{1,2\ldots ,s\right\},i\neq j}^{t}aa_{ij}x_{ik}x_{jk}+e_{h}+\sum_{i=1}^{s}ae_{hi}u_{hik}+\sum_{i,j\in \left\{1,2\ldots ,s\right\},i\neq j}^{t}aae_{hij}u_{hijk}+\epsilon_{hkm} \end{array}$$

where, *μ* is the population mean; *a_i_* is the additive effect of the *i*-th QTL with coefficient *x_ik_*, fixed effect; *aa_ij_* is the additive-by-additive epistatic effect of the *i*-th QTL and the j-th QTL with coefficient *x_ik_x_jk_*, fixed effect; *e_h_* is the main effect of the *h*-th environment, random effect, $e_{h}\sim \left(0,\sigma_{E}^{2}\right)$ ; *ae_hi_* is the additive-by-environment interaction effect of the *i*-th QTL and the *h*-th environment with coefficient *u_hik_* (=*x_ik_*)random effect, $ae_{hi}\sim \left(0,\;\sigma_{A_{i}E}^{2}\right)$ ; *aae_hij_* the interaction effect of the *aa_ij_* and the h-th environment with coefficient *u_hijk_* (=*x_ik_x_ik_*), random effect, $aae_{hij}\sim \left(0,\sigma_{AA_{ij}E}^{2}\right)$ ; and *ε_hkm_* is the residual effect of the individual, random, $\epsilon_{hkm}\sim \left(0,\;\sigma_{\epsilon }^{2}\right)$ .

*QTLNetwork* 2.0 software were employed to detect possible QTLs by the mixed-linear-model-based composite interval mapping (MCIM) method (Yang et al., 2008). One- and two-dimensional genome scans for QTLs were performed using a 5 cM testing window, a 0.5 cM walk speed and a 5 cM filtration window size. To control the experiment-wise type I error rate, a critical *F*-value based on the Henderson III method was determined by the permutation test with 1,000 times for each tested locus at the significance level of 0.05. Based on the significant QTL, A QTL full model was established and used to estimate each parameter based on the samples generated by Markov chain Monte Carlo (MCMC) with 20,000 Gibbs sampler iterations.

### 2.8 Candidate gene prediction

QTL intervals, determined by two adjacent markers in linkage map, were dissected for prediction of candidate gene(s) associated with the respective QTLs by bioinformatic analysis. Candidate genes in QTL regions and their corresponding protein sequence were selected from the annotation of K326 reference genome sequence (https://solgenomics.net/ftp/genomes/Nicotiana_tabacum/edwards_et_al_2017/annotation/Nitab-v4.5_gene_models_Scf_Edwards2017.gff) (https://solgenomics.net/ftp/genomes/Nicotiana_tabacum/edwards_et_al_2017/annotation/Nitab-v4.5_proteins_Edwards2017.fasta). The function of candidate genes was retrieved from the database UniProtKB/Swiss-Prot (ftp://ftp.ebi.ac.uk/pub/databases/uniprot/knowledgebase/uniprot_sprot.fasta.gz) using Protein BLAST (BLASTp) provided by NCBI, and selected on basis of E value = 1E−5.

## 3 Results

### 3.1 Phenotypic performance of six agronomic traits

**Figure 1:**
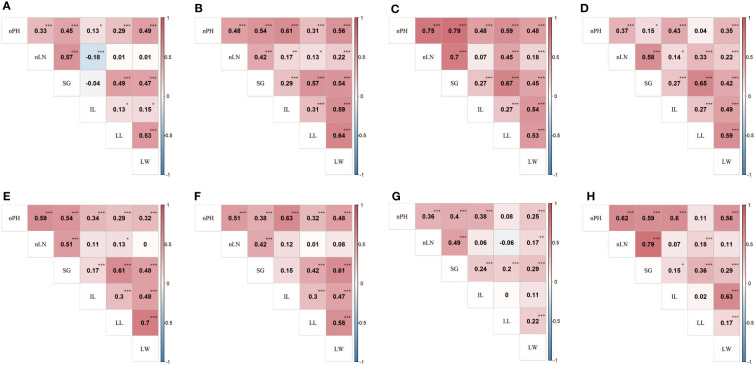
Phenotypic and genetic correlations between traits in the RIL population. Heat map **(A-G)** showed phenotypic correlation coefficients between six traits in E1 (2018 Shilin) - E7 (2021 Shilin) in turn, the heat map **(H)** showed genetic correlation coefficients between six traits. *,**,*** denote significance level at 0.05, 0.01 and 0.005, respectively. Traits abbreviations are same as those in **Table 1**.

For the six agronomic traits, the estimated heritability fluctuated from 12.57% (LL) to 57.72% (nLN), indicating that traits like nLN and nPH were more stable across environments, compared with the traits like LL and LW (**Table 1**). Most phenotypic correlations of the traits exhibited positive direction and reached statistically significant (*α*=0.05) (**Figures 1A–G**), while the correlation coefficient between LN and IL showed negative in E1 (**Figure 1A**), so as LN and LL in E7 (**Figure 1G**). The overall pattern fluctuated slightly and remained consistent in seven environments. The genetic correlation coefficients, which were calculated using the estimated genotypic values, exhibited the highest 0.79 between nLN and SG, followed by 0.63 between IL and LW, and a series of correlations between nPH and the other traits (apart from LL) over 0.5 (**Figure 1H**). Especially, we found that the phenotypic correlation between nLN and SG were shown relatively high in all seven environments, the high underlying genetic correlation might explain for it.

**Table 1:** Analysis of variance for six traits in seven environments.

Trait ^a^	Variance Components (σ^2^)^b^				Heritability ^c^ (%)
	$\sigma_{g}^{2}$	$\sigma_{e}^{2}$	$\sigma_{ge}^{2}$	$\sigma_{g}^{2}$	
nPH	318.45 (33.01)	402.76 (233.48)	33.99 (7.29)	358.67 (7.29)	44.78
nLN	9.98 (0.96)	2.87 (1.68)	0.30 (0.14)	7.01 (0.14)	57.72
SG	0.45 (0.05)	0.82 (0.48)	0.08 (0.01)	0.65 (0.01)	38.14
IL	0.12 (0.01)	0.48 (0.28)	0.04 (0.01)	0.26 (0.01)	29.69
LL	5.27 (0.97)	34.06 (19.73)	6.13 (0.68)	30.53 (0.68)	12.57
LW	3.88 (0.50)	10.17 (5.90)	1.70 (0.22)	10.33 (0.22)	24.40

a Traits abbreviations: nPH for natural plant height; nLN for natural leaf number; SG for stem girth; IL for inter-node length, LL for length of the largest waist leaf; LW for width of the largest waist leaf.

b Variance Components (σ^2^): $\sigma_{g}^{2}$ is for genotypic variance, $\;\sigma_{e}^{2}$ for environmental variance, $\sigma_{ge}^{2}$ for genotype-by-environment interaction variance, $\;\sigma_{\epsilon }^{2}$ for error variance.

c Heritability: $H^{2}=\sigma_{g}^{2}/\left(\sigma_{g}^{2}+\;\sigma_{ge}^{2}+\sigma_{\epsilon }^{2}\right)$ . Numbers in brackets were standard errors of predicted variance components.

### 3.2 Genetic linkage map

**Figure 2:**
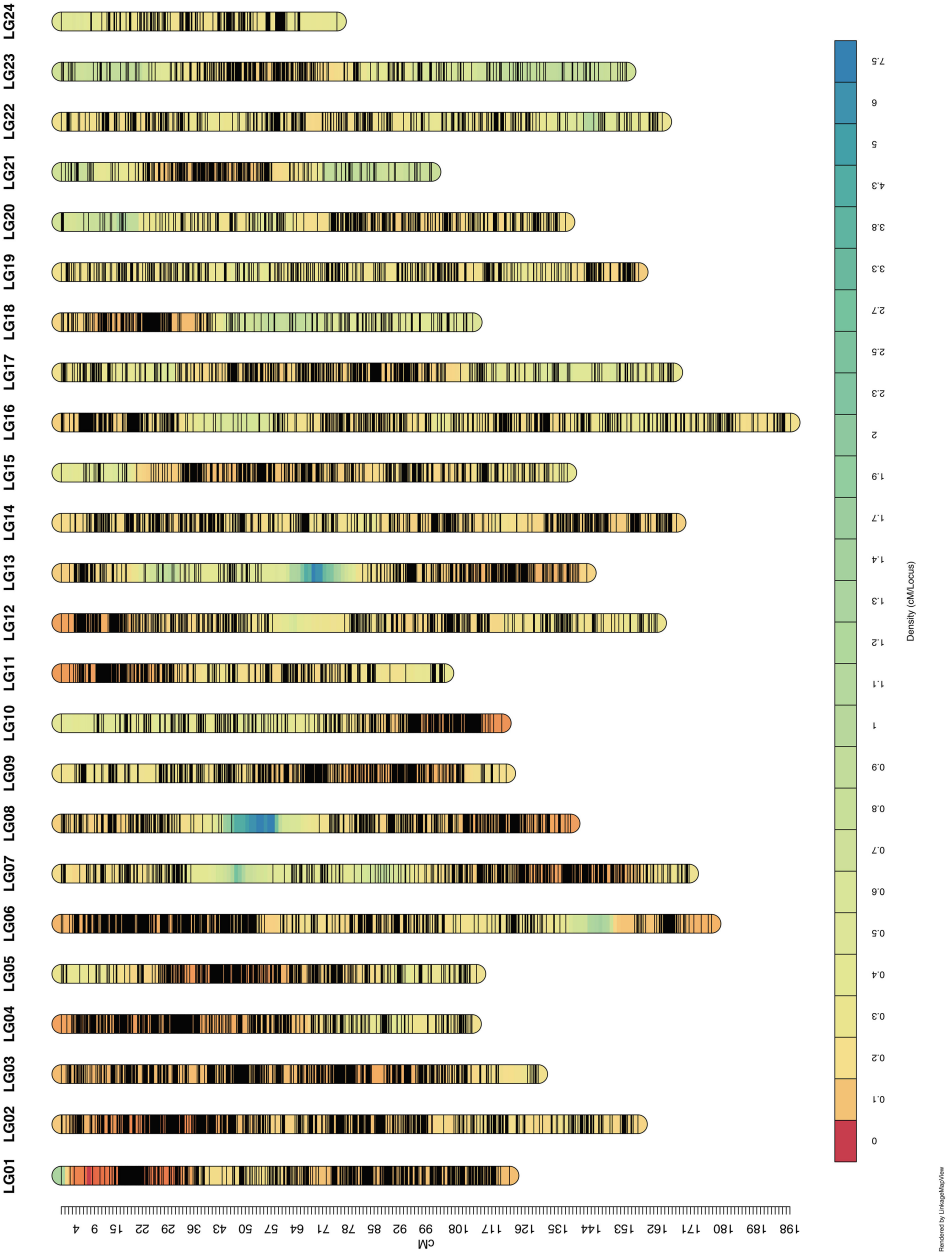
Linkage maps based on the reference genome. The linkage maps were constructed with a total of 46,324 markers. A total length of 3334.88 cM was mapped with 7,107 bin markers, and average distance was 0.469 cM.

We finally obtained 46,324 markers to generate a high-resolution genetic linkage map that contained 7,107 bin markers (defined as the marker with least genotype missing rate when there were multiple markers at the same genetic distance) mapped on 24 LGs and covered 3334.88 cM with an average genetic distance of 0.469cM (**Figure 2** and **Table 2**). The length of the LGs ranged from 74.94 cM to 198.21 cM, of which the shortest was LG24 containing 122 bin markers (0.614 cM/locus) and the longest was LG16 holding 406 bin markers (0.488 cM/locus). The number of bin markers on each LG varied from 122 to 436. Thus far, this genetic linkage map harboring 46,324 markers (7,107 bin markers) is the highest-resolution map reported in tobacco. Considering of the size of RIL population, the distribution density of the markers and the limitation of the software for QTL mapping, we then removed markers with adjacent distance less than 1cM, and retained 2,139 bin markers containing 1,918 SNPs, 76 indels and 145 SSR markers harboring on the 24 LGs for the subsequent QTL analysis (**Supplementary Table 2**).

**Table 2:** Summary of three types of markers in SNP-indel-SSR linkage map.

Linkage group	Number				Bin marker	Length (cM)	Average Length (cM)	Max gap (cM)
	Marker	SNP	Indel	SSR				
LG1	5,082	4,874	114	94	339	121.79	0.359	3.51
LG2	4,708	4,422	232	54	436	156.67	0.359	2.96
LG3	3,156	2,939	179	38	368	129.60	0.352	7.82
LG4	2,975	2,819	120	36	316	111.64	0.353	4.07
LG5	2,877	2,698	141	38	274	112.76	0.412	3.51
LG6	2,860	2,669	78	113	415	176.70	0.426	18.72
LG7	2,349	2,156	119	74	330	170.57	0.517	22.20
LG8	2,004	1,892	77	35	286	138.38	0.484	23.79
LG9	1,976	1,825	89	62	305	120.90	0.396	4.81
LG10	1,968	1,826	44	98	259	119.67	0.462	8.95
LG11	1,885	1,726	100	59	223	104.14	0.467	6.87
LG12	1,879	1,742	91	46	322	161.86	0.503	19.57
LG13	1,537	1,409	104	24	245	142.77	0.583	27.58
LG14	1,528	1,416	93	19	375	167.20	0.446	4.26
LG15	1,504	1,416	79	9	289	137.52	0.476	6.12
LG16	1,292	1,203	59	30	406	198.21	0.488	3.33
LG17	1,246	1,170	63	13	339	166.31	0.491	5.93
LG18	1,062	993	61	8	198	111.85	0.565	4.07
LG19	894	828	49	17	307	156.86	0.511	3.15
LG20	887	812	50	25	249	136.98	0.550	5.56
LG21	878	829	44	5	179	100.61	0.562	4.82
LG22	817	755	47	15	281	163.35	0.581	4.62
LG23	640	587	33	20	244	153.60	0.630	5.00
LG24	320	295	20	5	122	74.94	0.614	10.10
**Total**	46,324	43,301	2,086	937	7,107	3334.88	0.469	27.58

### 3.3 Additive and additive-by-environment interaction effects

According to the critical F-values to declare QTLs with significant single locus effects (called single-locus QTL hereafter) or to declare epistatic QTLs with significant epistatic effects (called epistatic QTL hereafter), a total of 70 QTLs consisting of 33 single-locus QTLs (**Table 3**) and 37 epistatic QTLs (**Table 4**) were mapped on 18 LGs, of which, 5 single-locus QTLs and 4 epistatic QTLs were detected for IL, 7 and 4 for LL, 6 and 5 for LW, 4 and 11 for nLN, 7 and 6 for nPH, 4 and 7 for SG, respectively (**Supplementary Table 3**). LG6 contained the most QTLs (19 QTLs), followed by LG4 (8 QTLs) and LG16 (7 QTLs), while LG11, LG13, LG19 contained the fewest QTLs (only 1 QTL on each).

**Table 3:** Effects and heritability of additive QTLs and additive-by-environment interaction detected for six traits.

Trait	QTL	*a* ^a^				*ae* ^b^				$h_{a}^{2}$ (%)^c^	$h_{ae}^{2}$ (%)^d^
			*ae_1_*	*ae_2_*	*ae_3_*	*ae_4_*	*ae_5_*	*ae_6_*	*ae_7_*		
nPH	*qnPH2*	-4.4467^***^								1.81	0
	*qnPH4*	-4.1185^***^								2.11	0
	*qnPH6-2*	-4.5539^***^	7.1966^***^	-13.0281^***^	-7.8158^***^	5.4854^***^	6.5205^***^			4.21	4.40
	*qnPH6-5*	-14.2296^***^				-4.5554^***^			4.9158^***^	12.68	0.64
	*qnPH11*	-3.3560^***^								0.76	0
	*qnPH17*	2.2404^***^								0.69	0
	*qnPH24*	3.4318^***^				5.0398^***^				1.01	0.73
nLN	*qnLN4-3*	-0.3545^***^								1.33	0
	*qnLN6-1*	-2.5516^***^			-0.6018^***^	0.3506^*^			0.6781^***^	34.80	1.30
	*qnLN6-3*	-1.7257^***^							0.5102^***^	5.04	0.26
	*qnLN17*	0.3695^***^								0.88	0
SG	*qSG6-1*	-0.3731^***^		-0.2836^***^		0.1666^***^	-0.3127^***^	0.2548^***^		10.34	1.92
	*qSG6-2*	-0.3545^***^				-0.0993^*^				2.51	0.26
	*qSG12-1*	-0.1400^***^								1.19	0
	*qSG24*	0.1506^***^				0.2194^***^				1.10	0.69
IL	*qIL4*	-0.1351^***^								1.82	0
	*qIL6-1*	0.0842^***^	0.0847^*^			0.0699^*^			-0.1664^***^	0.10	0.96
	*qIL6-3*	-0.2430^***^	0.1703^***^			-0.0929^*^				4.80	0.72
	*qIL10-1*	0.0658^***^								0.86	0
	*qIL24*	0.1110^***^								0.96	0
LL	*qLL4-2*	0.4301^***^			0.9532^***^					0.34	0.64
	*qLL6-1*	-0.4552^***^		-2.3763^***^		1.1722^***^	-2.4072^***^	2.6123^***^		0.40	2.84
	*qLL6-2*	1.3393^***^								1.67	0
	*qLL7*	0.6976^***^								0.67	0
	*qLL12*	-1.3666^***^								1.32	0
	*qLL13*	-0.8684^***^								0.83	0
	*qLL24*	0.8142^***^				1.4289^***^			-0.8103^*^	0.68	0.95
LW	*qLW4*	-0.8932^***^								2.25	0
	*qLW6-1*	0.4723^***^		-0.8015^***^	0.6836^***^	0.6541^***^	-0.7843^***^			0.98	1.42
	*qLW6-2*	-1.5662^***^				-0.7893^***^			0.4377^*^	6.05	0.43
	*qLW8-1*		-0.4547^*^		0.8887^***^	0.8002^***^		-0.8092^***^		0	1.18
	*qLW10*	0.7344^***^				0.5197^*^				0.72	0.45
	*qLW24*	0.6367^***^				0.6755^***^				1.60	0.71

a a, additive effect.

b ae, additive-by-environment interaction effects, of which ae_1_ denotes the interactions between a and environment E1.

c h^2^ heritability; $h_{a}^{2}$ : the proportion of phenotypic variance explained by the additive QTL.

d $h_{ae}^{2}$ , the proportion of phenotypic variance explained by the additive-by-environment interaction.

The critical F-values to declare QTL with significant single locus effects are 4.1 for nPH, nLN, SG, LL, LW, and 4.0 for IL, respectively.

^*^, ^**^, ^***^ denote significance level at 0.05, 0.01 and 0.005, respectively. Abbreviations of traits are same as those in **Table 1**.

**Table 4:** Effects and heritability of additive-by-additive epistatic QTLs and epistasis-by-environment interaction detected for six traits.

Trait	QTL*_i_*	QTL*_j_*	*aa* ^a^	*aae* ^b^							$h_{aa}^{2}$ (%)^c^	$h_{aae}^{2}$ (%)^d^
				*aae*_1_	*aae*_2_	*aae*_3_	*aae*_4_	*aae*_5_	*aae*_6_	*aae*_7_		
nPH	*qnPH6-1*	*qnPH8*	1.3663^**^								0.65	0
	*qnPH6-3*	*qnPH8*	1.8767^***^						-3.0289^*^		0.06	0.17
	*qnPH6-4*	*qnPH16-1*	-1.6932^***^								0.11	0
	*qnPH6-4*	*qnPH16-2*	3.0415^***^								0.18	0
nLN	*qnLN2*	*qnLN4-2*	-0.229^***^			-0.6456^***^		0.4095^*^	-0.4804^*^		0.14	0.88
	*qnLN4-1*	*qnLN12*	-0.1853^**^			-0.4852^***^					0.09	0.58
	*qnLN6-2*	*qnLN6-4*	0.4692^***^						-0.4386^*^		0.97	0.20
	*qnLN6-2*	*qnLN6-5*	0.3497^***^								0.04	0
	*qnLN15-1*	*qnLN18-2*	0.1831^**^								1.10	0
	*qnLN15-2*	*qnLN18-1*	0.3846^***^							-0.8027^***^	0.23	0.36
SG	*qSG12-2*	*qSG16-3*	-0.0743^***^								0.46	0
	*qSG12-3*	*qSG16-4*		-0.0998^*^							0	0.18
	*qSG16-1*	*qSG19*	0.1363^***^								0.30	0
	*qSG16-2*	*qSG19*			-0.152^*^			-0.1912^***^			0	0.30
IL	*qIL6-2*	*qIL10-2*	0.0935^***^	-0.0952^**^				0.0738^*^			0.48	0.38
	*qIL8*	*qIL16*	-0.0525^***^			-0.1641^***^				0.117^***^	0.42	0.97
LL	*qLL2*	*qLL9-2*				-1.1025^***^					0	0.48
	*qLL4-1*	*qLL9-1*	-0.5796^***^			-1.3212^***^					0.48	0.66
LW	*qLW2*	*qLW22-1*	0.4505^***^				-0.3427^*^				0.10	0.65
	*qLW2*	*qLW22-2*	-0.6848^***^								0.39	0
	*qLW7*	*qLW8-2*				0.9596^***^					0	0.91

a aa, additive-by-additive epistatic effect.

b aae, epistasis-by-environment interaction effects, of which aae_1_ denotes the interaction between aa and environment E1.

c h^2^, heritability; $h_{aa}^{2}$ : the proportion of phenotypic variance explained by the additive-additive epistatic effects.

d $h_{aae}^{2}$ , the proportion of phenotypic variance explained by the epistasis-by-environment interaction.

The critical F-value to declare epistatic QTLs with significant epistatic effects is 3.5 for nPH, 3.6 for nLN, 3.2 for SG, 3.1 for IL, 3.2 for LL and 3.3 for LW.

^*^, ^**^, ^***^ denote significance level at 0.05, 0.01 and 0.005, respectively. Abbreviations of traits are the same as **Table 1**.

Totally, 33 QTLs with additive (*a*) main effects and/or additive-by-environment interaction (*ae*) effects were detected for six traits. Most of the QTLs exhibited small additive effects, which were regarded as minor-effect QTLs, and about 70% of them explained less than 2% of the phenotypic variance (**Table 3**). The average proportion of phenotypic variance explained by a QTL ( $h_{a}^{2}$ ) was 3.23%. For LL, there were seven QTLs contributing small additive effects to phenotypic variation, indicating that the trait LL was under control of the minor-effect polygenes.

Nevertheless, there were seven QTLs with relatively large effects found in five traits, namely, *qnLN6-1* ( $h_{a}^{2}$ = 34.80%), *qnPH6-5* ( $h_{a}^{2}$ = 12.68%), *qSG6-1* ( $h_{a}^{2}$ =10.34%), *qLW6-2* ( $h_{a}^{2}$ = 6.05%), *qnLN6-3*( $h_{a}^{2}$ = 5.04%), *qIL6-3* ( $h_{a}^{2}$ = 4.80%) and *qnPH6-2* ( $h_{a}^{2}$ = 4.21%; **Table 3**), which played a major role in determining the variation of the corresponding traits. Intrinsically, we speculated that traits like nLN, nPH, SG, LW and IL were controlled by one or two major QTL(s)/gene(s). It was interesting that all seven major QTLs were distributed in two regions on LG6, wherein *qnLN6-1*, *qSG6-1* and *qnPH6-2* being mapped together on the position of 14.3 cM, whereas *qLW6-2* and *qnPH6-5* on the position of 166.3 cM; and the homozygous genotypes of the alleles from the parent K326 (*QQ*) at these seven QTLs all contributed negative effects (i.e., decreasing the trait value); in contrast, those of the alleles from the parent Y3 (*qq*) contributed positive effects (**Supplementary Table 4**). It was in accordance with the known fact that Y3 was an elite variety in agronomic performance.

Besides, some regions of the linkage groups harbored more than one QTL for different traits (**Supplementary Table 5**), which was in agreement with the significant genetic correlations estimated between studied traits (**Figure 1**). For example, *qnLN6-1*and *qSG6-1*were located exactly on the same genetic position of the linkage map, so as *qnLN6-3*and *qSG6-2*, which verified the existence of stronger genetic correlation up to 0.79 between nLN and SG.

Although significant additive by environment interaction effects (ae) were detected for more than half of QTLs, their contributions to phenotypic variation ( $h_{ae}^{2}$ ) were lower (less than 1%). However, a few *ae* effects were relatively large, for instance, the interaction between *qnPH6-2* and five (E1-E5) environments accounted for 4.40% of the phenotypic variance (**Table 3**). It was known to all that QTLs with no significant genotype-by-environment interaction effects will be important for breeding environmentally stable varieties. However, QTLs with relatively large effects interacted with environments more or less. Moreover, most of the *ae* effects exhibited the same (positive or negative) effect direction as those of the main effects of the QTLs, while some of them showed the opposite. For example, *qLL6-1* decreased the length of the largest leaf, whereas its interaction effects with environment E4 and E6 increased the value of the trait.

### 3.4 Additive-by-additive epistasis and epistasis-by-environment interaction effects

Two-dimensional genome scan detected 21 digenic epistatic QTL pairs with additive-by-additive epistasis (*aa*) effects and/or epistasis-by-environment interaction (*aae*) effects for six traits, in which 8 pairs had *aa* effects only, 4 pairs had *aae* effects only and 9 pairs had both *aa* and *aae* effects. However, 21 epistatic QTLs which involved 37 loci contributed insignificant additive-additive epistasis effects (**Table 4**). What’s more, the *aae* may performed oppositely in different environments. For example, the environment-specific epistatic effect (*aae*) between *qnLN2* and *qnLN4-2* in E3/E6 was in the same direction with that of the *aa* effect, while its interaction with E5 effected in opposite. Both *aa* and *aae* effects contributed limited magnitude to phenotypic variance, mostly less than 1% (**Table 4**).

Overall, for the traits except nLN, environmental (E) effects explained the biggest part of the phenotypic variance which ranged from 31.65% to 47.82%, followed by genetic (G) main effects and genotype-by-environment interaction (GE) effects. While for nLN, the genetic effect was the major component of the phenotypic variance, wherein the additive effect of *qnLN6-1* contributed a large part to the total variation (**Supplementary Table 6**).

### 3.5 Breeding potential of predicted lines

In terms of general genetic main effects, P2 (Y3) performed much better than P1 (K326) in most agronomic traits ignoring gene by environment interaction (**Supplementary Table 7**), showing the excellent characters of Y3 in agronomic traits. For example, the predicted general genetic value of nPH was 34.2562 for Y3 and -20.4412 for K326. The general superior lines (GSL) were designed by all QTL genotypes with maximized genetic values (**Supplementary Table 4**), which would provide guidance in improving agronomic traits. The genetic values of the GSL achieved 44.3546 for nPH, 6.8022 for nLN, 1.2289 for SG, 0.7852 for IL, 6.5509 for LL and 5.4382 for LW.

### 3.6 Prediction of candidate gene in major QTLs

On the one hand, the genetic correlations between some traits were especially high with coefficients over 0.5 (**Figure 1**), indicating that there might exist pleiotropic genes accounting for it. On the other hand, six major QTLs of these traits happened to be mapped in two regions, where pleiotropic genes might harbor and it was more possible to find potential candidate genes from these regions.

The *qnLN6-1* $\left(h_{a}^{2}=34.80\%\right)$ , q*SG6-1* $\left(h_{a}^{2}=10.34\%\right)$ and *qnPH6-2* $\left(h_{a}^{2}=4.21\%\right)$ were mapped on the same genetic position (**Supplementary Table 5**), closely correlated with the markers PT50965 and SNP_0386058_322; they were determined to be located on the region from 8,165,328 bp to 8,979,225 bp on Nt16 based on the K326 reference genome reported by Edwards et al. (2017). Comparative mapping of the K326 reference genome predicted the existence of a total of 19 genes from 81.65 Mb to 89.79 Mb on Nt16 (**Supplementary Table 8**). Among them, one gene *Nt16g00284.1* encoding a protein with high homology to *FRAGILE FIBER3* (*FRA3*) in *Arabidopsis thaliana* (Zhong et al., 2004) was considered as the candidate pleiotropic gene for trait nLN, SG and nPH. *FRA3* encodes a type II 5PTase, plays an essential role in the secondary wall synthesis in fiber cells and xylem vessels and is highly expressed in fiber cells and vascular tissues in stems.

Another group of major-effect QTLs, *qnPH6-5* $\left(h_{a}^{2}=12.68\%\right)$ , *qLW6-2* $\left(h_{a}^{2}=6.05\%\right)$ and *qIL6-3* $\left(h_{a}^{2}=4.80\%\right)$ , were found to be at the region between markers TM23680 and SNP_0000403_34752 simultaneously. A total of 173 genes were annotated on this target region occupied from 24,110,108 bp to 39,896,452 bp on Nt16 (**Supplementary Table 8**). We finally screened out three candidate genes: *Nt16g00767.1* gene, which encodes a protein highly homologous to *BRASSINOSTEROID INSENSITIVE 1* (*BRI1*) in *Arabidopsis thaliana* (Domagalska et al., 2007); *Nt16g00853.1* gene, which encodes a protein with high homology to *BRASSINOSTEROID-RESPONSIVE RING-H2* (*BRH1*) in *Arabidopsis thaliana*(Wang et al., 2018); *Nt16g00877.1* gene, which encodes a protein highly homologous to *SOSEKI2* (*SOK2*) in *Arabidopsis thaliana*(Finnegan et al., 2004). *BRI1* appears to be involved in the autonomous pathway that regulates the transition to flowering, primarily through its effects on FLOWERING LOCUS C (FLC) expression levels; *BRH1* mediates BRs signaling pathway, which also influence FLC expression; *SOK2* is one part of a three-gene cluster containing FLC, UFC and DFC, all of three genes are associated with FLC. Encoding a MADS-box transcription factor, FLC is a major repressor of flowering in Arabidopsis (Deng et al., 2011), therefore, we speculated that the extension of vegetative stage might prolong the period of transition and provide more time for stems and leaves to thrive.

## 4 Discussion

We have known that the genetic mechanisms underlying important agronomic traits in tobacco are not well-characterized owing to the genome complexity and limited molecular resources. As SNP is one of the most abundant forms of genetic variation (Ganal et al., 2009), a high-resolution genetic map, containing 45,081 SNPs markers covered 3486.78 cM with an average genetic distance of 0.495 cM, has been constructed by whole genome sequencing in tobacco (Tong et al., 2020). In this study, we further took informative genetic markers like indels and SSRs into account to explore the genetic architecture of agronomic traits in tobacco, which has been proved to be effective by numerous studies in various plants. While SSR markers have been contributing to the studies of tobacco over the past few decades (Bindler et al., 2007; Moon et al., 2009; Bindler et al., 2011; Tong et al., 2012; Yang et al., 2020), indels have not been investigated until recent years. In order to achieve more reliable results under a more thorough design, we finally merged 43,301 SNPs, 2086 indels and 937 SSRs together to construct an integrated higher-density linkage map covering 3334.88 cM with an average genetic distance of 0.469cM. Moreover, the QTL analysis based on this new linkage map indicated the indispensability of SSR markers (e.g. PT50965 and PT30412) in determining the position of QTLs and exploring genetic architectures of agronomic traits (**Supplementary Table 3**).

Considering of involvement of gene-gene, gene-environment interaction in the genetic variation of complex traits, we used a full-QTL model to integrate the effects of additive QTLs, epistasis and GE interactions into one mapping system provided by QTLNetwork2.0, which has not been taken into consideration in most QTL studies of agronomic traits in tobacco. The QTL main effects are treated as fixed, which is valid across different environment; while, the QTL-environment interaction effects as random, which are deviation from the average effect of QTL effects across all environments, zero summation of which is assumed. Therefore, some positive or negative QTL-environment interaction effects (*ae, aae*) would be observed in results. For six traits, a total of 17 epistasis QTL pairs were detected. These results proved that non-additive epistatic interactions are an important contributor to trait variation, which has been documented by numerous studies before (Kim and Rieseberg, 2001; Xing et al., 2002; Kroymann and Mitchell-Olds, 2005; Malmberg et al., 2005). What’s more, we also identified some environment-specific QTL effects (*ae* and *aae*) under multiple environments, which could be utilized in genetic improvement of tobacco to breed environment-specific or strong environment adaptability elite varieties in future breeding program. Compared with those models with QTL additive effects only, this model indeed offered us new insights into understanding of genetic architecture of quantitative traits. However, this study found that the additive effects were the main force accounting for heritability and provided guidance for further studies and utilization of the QTLs, such as, fine mapping, cloning and marker-assisted selection. Such as *qnPH6-5, qnLN6-1, qnLN6-3, qSG6-1*, *qIL6-3* and *qLW6-2*, they contributed relatively larger additive effects compared with additive-environment interaction effects, indicating selection on them is valid across environments; while, *qnPH6-2* exhibited similar magnitude of *a* and *ae* effects, indicating it could be selectively utilized in different environment. As we conjectured that nLN, nPH, SG, IL, LW were controlled by several major effect QTLs/genes, especially for nLN, whose main effects were considerable compared with that of environment interaction; therefore, these major QTLs are promising and should be paid more attention in following-up utilization in breeding higher-yield varieties.

Based on the results of QTL mapping, mining candidate genes controlling the traits could be confined to the regions of QTLs. However, based only on preliminary mapping population (RILs) like our study, it is still a challenge to distinguish candidate genes of target trait from a lot of annotated genes in QTL regions. Therefore, further studies such as fine mapping using secondary mapping populations is required to narrow down the target QTL. Including single segment substitution lines (SSSLs), near isogenic lines (NILs) and chromosome segment substitution lines (CSSLs), secondary mapping populations are identical to the recurrent parent for all trait loci except the target QTLs, which can eliminate the interference of genetic background and improve the sensitivity and accuracy of miming and prediction of candidate genes in QTL. What’s more, functional validation of candidate genes with molecular biology techniques on the levels of gene expressions, proteins and metabolites should be implemented to understand the genetic regulation mechanism of traits, which is valuable to guide us for designing superior genotype with better performance of agronomic traits.

## Data availability statement

The data presented in the study are deposited in the European Nucleotide Archive (ENA) repository, accession number PRJEB59363 (https://www.ebi.ac.uk/ena/browser/view/PRJEB59363).

## Author contributions

ZT and BX designed the study; ZT and MX wrote the manuscript; MX, QZ, FL, TZ, YL and HX analyzed the data; ZT, DF, XC and BX conducted the field experiment; HX and BX revised the manuscript. All authors contributed to the manuscript and approved the submitted version.

## References

[B1] Bao F. Du D. An Y. Yang W. Wang J. Cheng T.. (2017). Overexpression of prunus mume dehydrin genes in tobacco enhances tolerance to cold and drought. Front. Plant Sci. 8. doi: 10.3389/fpls.2017.00151 PMC5293821 28224001

[B2] Bindler G. Plieske J. Bakaher N. Gunduz I. Ivanov N. van der Hoeven R.. (2011). A high density genetic map of tobacco (Nicotiana tabacum l.) obtained from large scale microsatellite marker development. Theor. Appl. Genet. 123, 219–230. doi: 10.1007/s00122-011-1578-8 21461649 PMC3114088

[B3] Bindler G. van der Hoeven R. Gunduz I. Plieske J. Ganal M. Rossi L.. (2007). A microsatellite marker based linkage map of tobacco. Theor. Appl. Genet. 114, 341–349. doi: 10.1007/s00122-006-0437-5 17115128

[B4] Cheng L. Chen X. Jiang C. Ma B. Ren M. Cheng Y.. (2019). High-density SNP genetic linkage map construction and quantitative trait locus mapping for resistance to cucumber mosaic virus in tobacco (Nicotiana tabacum l.). Crop J. 7, 539–547. doi: 10.1016/j.cj.2018.11.010

[B5] Cheng L. Yang A. Jiang C. Ren M. Zhang Y. Feng Q.. (2015). Quantitative trait loci mapping for plant height in tobacco using linkage and association mapping methods. Crop Sci. 55, 641–647. doi: 10.2135/cropsci2014.05.0404

[B6] Covarrubias-Pazaran G. (2016). Genome-assisted prediction of quantitative traits using the r package sommer. PloS One 11. doi: 10.1371/journal.pone.0156744 PMC4894563 27271781

[B7] Dixit S. Singh A. Sandhu N. Bhandari A. Vikram P. Kumar A. (2017). Combining drought and submergence tolerance in rice: marker-assisted breeding and QTL combination effects. Mol. Breed. 37. doi: 10.1007/s11032-017-0737-2 PMC5670188 29151804

[B8] Deng W. Ying H. Helliwell C. A. Taylor J. M. Peacock W. J. Dennis E. S. (2011). FLOWERING LOCUS C (FLC) regulates development pathways throughout the life cycle of Arabidopsis. Proc Natl Acad Sci U S A 108, 6680–6685. doi: 10.1073/pnas.1103175108 PMC3081018 21464308

[B9] Drake-Stowe K. Bakaher N. Goepfert S. Philippon B. Mark R. Peterson P.. (2017). Multiple disease resistance loci affect soilborne disease resistance in tobacco (Nicotiana tabacum). Phytopathology 107, 1055–1061. doi: 10.1094/PHYTO-03-17-0118-R 28581342

[B10] Domagalska M. A. Schomburg F. M. Amasino R. M. Vierstra R. D. Nagy F. Davis S. J.. (2007). Attenuation of brassinosteroid signaling enhances FLC expression and delays flowering. Development 134, 2841–2850. doi: 10.1242/dev.02866 17611230

[B11] Edwards K. D. Fernandez-Pozo N. Drake-Stowe K. Humphry M. Evans A. D. Bombarely A.. (2017). A reference genome for nicotiana tabacum enables map-based cloning of homeologous loci implicated in nitrogen utilization efficiency. BMC Genomics 18. doi: 10.1186/s12864-017-3791-6 PMC5474855 28625162

[B12] Finnegan E. J. Sheldon C. C. Jardinaud F. Peacock W. J. Dennis E. S. (2004). A cluster of arabidopsis genes with a coordinate response to an environmental stimulus the observation that a cold treatment downregulates these two genes raises the question of whether there is a genome-wide downregulation of transcription in ver. Curr. Biol. 14, 911–916. doi: 10.1016/j 15186749

[B13] Food and Agriculture Organization of the United Nations. (2020). https://www.fao.org/faostat/en/#data/QCL

[B14] Ganal M. W. Altmann T. Röder M. S. (2009). SNP identification in crop plants. Curr. Opin. Plant Biol. 12, 211–217. doi: 10.1016/j.pbi.2008.12.009 19186095

[B15] Gong D. Huang L. Xu X. Wang C. Ren M. Wang C.. (2016). Construction of a high-density SNP genetic map in flue-cured tobacco based on SLAF-seq. Mol. Breed. 36. doi: 10.1007/s11032-016-0514-7

[B16] Hu Z. Lu S. J. Wang M. J. He H. Sun L. Wang H.. (2018). A novel QTL qTGW3 encodes the GSK3/SHAGGY-like kinase OsGSK5/OsSK41 that interacts with OsARF4 to negatively regulate grain size and weight in rice. Mol. Plant 11, 736–749. doi: 10.1016/j.molp.2018.03.005 29567449

[B17] Julio E. Denoyes-Rothan B. Verrier J. L. Dorlhac De Borne F. (2006). Detection of QTLs linked to leaf and smoke properties in nicotiana tabacum based on a study of 114 recombinant inbred lines. Mol. Breed. 18, 69–91. doi: 10.1007/s11032-006-9019-0

[B18] Kim S. C. Rieseberg L. H. (2001). The contribution of epistasis to species differences in annual sunflowers. Mol. Ecol. 10, 683–690. doi: 10.1046/j.1365-294X.2001.01203.x 11298979

[B19] Kroymann J. Mitchell-Olds T. (2005). Epistasis and balanced polymorphism influencing complex trait variation. Nature 435, 95–98. doi: 10.1038/nature03480 15875023

[B20] Lee Y. K. Kim I. J. (2018). Functional conservation of arabidopsis LNG1 in tobacco relating to leaf shape change by increasing longitudinal cell elongation by overexpression. Genes Genomics 40, 1053–1062. doi: 10.1007/s13258-018-0712-2 29949075

[B21] Leitch I. J. Hanson L. Lim K. Y. Kovarik A. Chase M. W. Clarkson J. J.. (2008). The ups and downs of genome size evolution in polyploid species of nicotiana (Solanaceae). in. Ann. Bot., 101(6), 805–814. doi: 10.1093/aob/mcm326 18222910 PMC2710205

[B22] Lewis R. S. Milla S. R. Kernodle S. P. (2007). Analysis of an introgressed nicotiana tomentosa genomic region affecting leaf number and correlated traits in nicotiana tabacum. Theor. Appl. Genet. 114, 841–854. doi: 10.1007/s00122-006-0482-0 17219207

[B23] Li H. (2013). Aligning sequence reads, clone sequences and assembly contigs with BWA-MEM. ArXiv 1303, 3997. doi: 10.48550/arXiv.1303.3997

[B24] Li S. Tian Y. Wu K. Ye Y. Yu J. Zhang J.. (2018). Modulating plant growth–metabolism coordination for sustainable agriculture. Nature 560, 595–600. doi: 10.1038/s41586-018-0415-5 30111841 PMC6155485

[B25] Liu Y. Wang L. Sun C. Zhang Z. Zheng Y. Qiu F. (2014). Genetic analysis and major QTL detection for maize kernel size and weight in multi-environments. Theor. Appl. Genet. 127, 1019–1037. doi: 10.1007/s00122-014-2276-0 24553962

[B26] Liu Y. J. Xiu Z. H. Meeley R. Tan B. C. (2013). Empty pericarp5 encodes a pentatricopeptide repeat protein that is required for mitochondrial RNA editing and seed development in maize. Plant Cell 25, 868–883. doi: 10.1105/tpc.112.106781 23463776 PMC3634694

[B27] Liu Y. Yuan G. Si H. Sun Y. Jiang Z. Liu D.. (2022). Identification of QTLs associated with agronomic traits in tobacco *via* a biparental population and an eight-way MAGIC population. Front. Plant Sci. 13. doi: 10.3389/fpls.2022.878267 PMC9207565 35734263

[B28] Mackay T. F. C. (2014). Epistasis and quantitative traits: Using model organisms to study gene-gene interactions. Nat. Rev. Genet. 15, 22–33. doi: 10.1038/nrg3627 24296533 PMC3918431

[B29] Ma J. M. Heim C. Humphry M. Nifong J. M. Lewis R. S. (2019). Genetic analysis of Phn7.1, a major QTL conferring partial resistance to phytophthora nicotianae in nicotiana tabacum. Mol. Breed. 39. doi: 10.1007/s11032-018-0923-x

[B30] Malmberg R. L. Held S. Waits A. Mauricio R. (2005). Epistasis for fitness-related quantitative traits in arabidopsis thaliana grown in the field and in the greenhouse. Genetics 171, 2013–2027. doi: 10.1534/genetics.105.046078 16157670 PMC1456117

[B31] McKenna A. Hanna M. Banks E. Sivachenko A. Cibulskis K. Kernytsky A.. (2010). The genome analysis toolkit: A MapReduce framework for analyzing next-generation DNA sequencing data. Genome Res. 20, 1297–1303. doi: 10.1101/gr.107524.110 20644199 PMC2928508

[B32] Moon H. S. Nifong J. M. Nicholson J. S. Heineman A. Lion K. van der Hoeven R.. (2009). Microsatellite-based analysis of tobacco (Nicotiana tabacum l.) genetic resources. Crop Sci. 49, 2149–2159. doi: 10.2135/cropsci2009.01.0024

[B33] Ouellette L. A. Reid R. W. Blanchard S. G. Brouwer C. R. (2018). LinkageMapView-rendering high-resolution linkage and QTL maps. Bioinformatics 34, 306–307. doi: 10.1093/bioinformatics/btx576 28968706 PMC5860205

[B34] Pagliano C. Bersanini L. Cella R. Longoni P. Pantaleoni L. Dass A.. (2017). Use of nicotiana tabacum transplastomic plants engineered to express a his-tagged CP47 for the isolation of functional photosystem II core complexes. Plant Physiol. Biochem. 111, 266–273. doi: 10.1016/j.plaphy.2016.12.009 27987471

[B35] Prat N. Guilbert C. Prah U. Wachter E. Steiner B. Langin T.. (2017). QTL mapping of fusarium head blight resistance in three related durum wheat populations. Theor. Appl. Genet. 130, 13–27. doi: 10.1007/s00122-016-2785-0 27662843 PMC5215227

[B36] Rastas P. (2017). Lep-MAP3: Robust linkage mapping even for low-coverage whole genome sequencing data. Bioinformatics 33, 3726–3732. doi: 10.1093/bioinformatics/btx494 29036272

[B37] Sierro N. Battey J. N. D. Ouadi S. Bakaher N. Bovet L. Willig A.. (2014). The tobacco genome sequence and its comparison with those of tomato and potato. Nat. Commun. 5. doi: 10.1038/ncomms4833 PMC4024737 24807620

[B38] Sierro N. Battey J. N. D. Ouadi S. Bovet L. Goepfert S. Bakaher N.. (2013). Reference genomes and transcriptomes of nicotiana sylvestris and nicotiana tomentosiformis. Genome Biol. 14. doi: 10.1186/gb-2013-14-6-r60 PMC3707018 23773524

[B39] Sun F. Wang X. Bonnard G. Shen Y. Xiu Z. Li X.. (2015). Empty pericarp7 encodes a mitochondrial e-subgroup pentatricopeptide repeat protein that is required for ccmFN editing, mitochondrial function and seed development in maize. Plant J. 84, 283–295. doi: 10.1111/tpj.12993 26303363

[B40] Thimmegowda G. C. Ramadoss S. K. Kaikala V. Rathinavelu R. Thamalampudi V. R. Dhavala V. N. C.. (2018). Whole genome resequencing of tobacco (Nicotiana tabacum l.) genotypes and high-throughput SNP discovery. Mol. Breed. 38. doi: 10.1007/s11032-018-0876-0

[B41] Tong Z. Xiao B. Jiao F. Fang D. Zeng J. Wu X.. (2016). Large-Scale development of SSR markers in tobacco and construction of a linkage map in flue-cured tobacco. Breed Sci. 66, 381–390. doi: 10.1270/jsbbs.15129 27436948 PMC4902457

[B42] Tong Z. Xiu Z. Ming Y. Fang D. Chen X. Hu Y.. (2021). Quantitative trait locus mapping and genomic selection of tobacco (Nicotiana tabacum l.) based on high-density genetic map. Plant Biotechnol. Rep. 15, 845–854. doi: 10.1007/s11816-021-00713-1

[B43] Tong Z. Yang Z. Chen X. Jiao F. Li X. Wu X.. (2012). Large-Scale development of microsatellite markers in nicotiana tabacum and construction of a genetic map of flue-cured tobacco. Plant Breed. 131, 674–680. doi: 10.1111/j.1439-0523.2012.01984.x

[B44] Tong Z. Zhou J. Xiu Z. Jiao F. Hu Y. Zheng F.. (2020). Construction of a high-density genetic map with whole genome sequencing in nicotiana tabacum l. Genomics 112, 2028–2033. doi: 10.1016/j.ygeno.2019.11.015 31760041

[B45] Tura H. Edwards J. Gahlaut V. Garcia M. Sznajder B. Baumann U.. (2020). QTL analysis and fine mapping of a QTL for yield-related traits in wheat grown in dry and hot environments. Theor. Appl. Genet. 133, 239–257. doi: 10.1007/s00122-019-03454-6 31586227 PMC7990757

[B46] Vijay V. Danehower D. A. Tyler S. Moon H. S. Lewis R. S. (2010). Analysis of a nicotiana tabacum l. genomic region controlling two leaf surface chemistry traits. J. Agric. Food Chem. 58, 294–300. doi: 10.1021/jf903256h 20014852

[B47] Wang G. F. Balint-Kurti P. J. (2016). Maize homologs of CCoAOMT and HCT, two key enzymes in lignin biosynthesis, form complexes with the NLR Rp1 protein to modulate the defense respons. Plant Physiol. 171, 2166–2177. doi: 10.1104/pp.16.00224 27208251 PMC4936554

[B48] Wang X. Bennetzen J. L. (2015). Current status and prospects for the study of nicotiana genomics, genetics, and nicotine biosynthesis genes. Mol. Genet. Genomics 290, 11–21. doi: 10.1007/s00438-015-0989-7 25582664

[B49] Wang X. Chen E. Ge X. Gong Q. Butt H. I. Zhang C.. (2018). Overexpressed BRH1, a RING finger gene, alters rosette leaf shape in arabidopsis thaliana. Sci. China Life Sci. 61, 79–87. doi: 10.1007/s11427-017-9133-8 28887625

[B50] Wang X. Pang Y. Zhang J. Zhang Q. Tao Y. Feng B.. (2014). Genetic background effects on QTL and QTL × environment interaction for yield and its component traits as revealed by reciprocal introgression lines in rice. Crop J. 2, 345–357. doi: 10.1016/j.cj.2014.06.004

[B51] Wu S. Guo Y. Adil M. F. Sehar S. Cai B. Xiang Z.. (2020). Comparative proteomic analysis by iTRAQ reveals that plastid pigment metabolism contributes to leaf color changes in tobacco (Nicotiana tabacum) during curing. Int. J. Mol. Sci. 21. doi: 10.3390/ijms21072394 PMC7178154 32244294

[B52] Wu Q. Wu X. Zhang X. Jiang C. Xiao B. Zhang Y.. (2014). Mapping of two white stem genes in tetraploid common tobacco (Nicotiana tabacum l.). Mol. Breed. 34, 1065–1074. doi: 10.1007/s11032-014-0097-0

[B53] Xiao B. Tan Y. Long N. Chen X. Tong Z. Dong Y.. (2015). SNP-based genetic linkage map of tobacco (Nicotiana tabacum l.) using next-generation RAD sequencing. J. Biol. Research-Thessaloniki 22. doi: 10.1186/s40709-015-0034-3 PMC4607152 26473145

[B54] Xing Y. Z. Tan Y. F. Hua J. P. Sun X. L. Xu C. G. Zhang Q. (2002). Characterization of the main effects, epistatic effects and their environmental interactions of QTLs on the genetic basis of yield traits in rice. Theor. Appl. Genet. 105, 248–257. doi: 10.1007/s00122-002-0952-y 12582526

[B55] Xiu Z. Sun F. Shen Y. Zhang X. Jiang R. Bonnard G.. (2016). EMPTY PERICARP16 is required for mitochondrial nad2 intron 4 cis-splicing, complex i assembly and seed development in maize. Plant J. 85, 507–519. doi: 10.1111/tpj.13122 26764126

[B56] Yang H. Geng X. Zhao S. Shi H. (2020). Genomic diversity analysis and identification of novel SSR markers in four tobacco varieties by high-throughput resequencing. Plant Physiol. Biochem. 150, 80–89. doi: 10.1016/j.plaphy.2020.02.023 32126511

[B57] Yang J. Hu C. Hu H. Yu R. Xia Z. Ye X.. (2008). QTLNetwork: Mapping and visualizing genetic architecture of complex traits in experimental populations. Bioinformatics 24, 721–723. doi: 10.1093/bioinformatics/btm494 18202029

[B58] Zhang Y. Guo X. Yan X. Ren M. Jiang C. Cheng Y.. (2018). Identification of stably expressed QTL for resistance to black shank disease in tobacco (Nicotiana tabacum l.) line beinhart 1000-1. Crop J. 6, 282–290. doi: 10.1016/j.cj.2017.12.002

[B59] Zhang X. Huang C. Wu D. Qiao F. Li W. Duan L.. (2017). High-throughput phenotyping and QTL mapping reveals the genetic architecture of maize plant growth. Plant Physiol. 173, 1554–1564. doi: 10.1104/pp.16.01516 28153923 PMC5338669

[B60] Zhong R. Burk D. H. Morrison W. H. Ye Z. H. (2004). FRAGILE FIBER3, an arabidopsis gene encoding a type ii inositol polyphosphate 5-phosphatase, is required for secondary wall synthesis and actin organization in fiber cells. Plant Cell 16, 3242–3259. doi: 10.1105/tpc.104.027466 15539468 PMC535871

[B61] Zimmerman J. L. Goldberg R. B. (1977). DNA Sequence organization in the genome of nicotiana tabacum. Chromosoma 59, 227–252. doi: 10.1007/BF00292780

